# Transcriptional profiling of multiple system atrophy cerebellar tissue highlights differences between the parkinsonian and cerebellar sub-types of the disease

**DOI:** 10.1186/s40478-020-00950-5

**Published:** 2020-06-03

**Authors:** Ignazio S. Piras, Christiane Bleul, Isabelle Schrauwen, Joshua Talboom, Lorida Llaci, Matthew D. De Both, Marcus A. Naymik, Glenda Halliday, Conceicao Bettencourt, Janice L. Holton, Geidy E. Serrano, Lucia I. Sue, Thomas G. Beach, Nadia Stefanova, Matthew J. Huentelman

**Affiliations:** 1 Neurogenomics Division, The Translational Genomics Research Institute, Phoenix, AZ USA; 2 Present Address: Department of Neurology, Center for Statistical Genetics, Gertrude H. Sergievsky Center, Columbia University Medical Center, 630 W 168th St, New York, NY 10032 USA; 3 Present address: Division of Biology and Biomedical Sciences, Molecular Genetics and Genomics Program, Washington University in St. Louis, St. Louis, MO 63110 USA; 4 The University of Sydney Brain and Mind Centre and Faculty of Medicine and Health, School of Medical Science, and Neuroscience Research Australia, Sydney, Australia; 5 Queen Square Brain Bank for Neurological Disorders and Department of Clinical and Movement Neurosciences, UCL Queen Square Institute of Neurology, London, UK; 6 Civin Laboratory of Neuropathology at Banner Sun Health Research Institute, Sun City, AZ 85351 USA; 7 Department of Neurology, Division of Neurobiology, Medical University of Innsbruck, Innsbruck, Austria

**Keywords:** Multiple system atrophy, RNA sequencing, Oligodendrocytes, Neurodegeneration

## Abstract

Multiple system atrophy (MSA) is a rare adult-onset neurodegenerative disease of unknown cause, with no effective therapeutic options, and no cure. Limited work to date has attempted to characterize the transcriptional changes associated with the disease, which presents as either predominating parkinsonian (MSA-P) or cerebellar (MSC-C) symptoms. We report here the results of RNA expression profiling of cerebellar white matter (CWM) tissue from two independent cohorts of MSA patients (*n* = 66) and healthy controls (HC; *n* = 66). RNA samples from bulk brain tissue and from oligodendrocytes obtained by laser capture microdissection (LCM) were sequenced. Differentially expressed genes (DEGs) were obtained and were examined before and after stratifying by MSA clinical sub-type.

We detected the highest number of DEGs in the MSA-C group (*n* = 747) while only one gene was noted in MSA-P, highlighting the larger dysregulation of the transcriptome in the MSA-C CWM. Results from both bulk tissue and LCM analysis showed a downregulation of oligodendrocyte genes and an enrichment for myelination processes with a key role noted for the *QKI* gene. Additionally, we observed a significant upregulation of neuron-specific gene expression in MSA-C and enrichment for synaptic processes. A third cluster of genes was associated with the upregulation of astrocyte and endothelial genes, two cell types with a key role in inflammation processes. Finally, network analysis in MSA-C showed enrichment for β-amyloid related functional classes, including the known Alzheimer’s disease (AD) genes, *APP* and *PSEN1*.

This is the largest RNA profiling study ever conducted on post-mortem brain tissue from MSA patients. We were able to define specific gene expression signatures for MSA-C highlighting the different stages of the complex neurodegenerative cascade of the disease that included alterations in several cell-specific transcriptional programs. Finally, several results suggest a common transcriptional dysregulation between MSA and AD-related genes despite the clinical and neuropathological distinctions between the two diseases.

## Introduction

Multiple-system atrophy (MSA) is a rare neurodegenerative disorder characterized by autonomic dysfunction, ataxia, and parkinsonism. The prevalence is estimated to be between 1.9 to 4.9 per 100,000 [10, 67]. The disease affects both sexes equally with onset typically in the sixth decade of life and with an average survival after diagnosis of less than 10 years [67]. There are no effective long-term therapeutic options for the MSA patient, and no cure.

MSA as a unifying diagnostic terminology was developed to encapsulate three neurological entities: striatonigral degeneration, olivopontocerebellar atrophy, and Shy-Drager syndrome [28, 55, 73, 74]. Two different clinical subtypes have been described based on the predominating motor features noted during the early stages of the disease: the MSA-P subtype (dominated by parkinsonism) and the MSA-C subtype (dominated by cerebellar ataxia). However, in the later stages of the disease, the phenotypic characteristics of both subtypes are typically noted in the patient [18]. A definitive diagnosis of MSA is obtained through autopsy confirmation of a high density of α-synuclein-containing protein aggregates, known as glial cytoplasmic inclusion (GCI) bodies, in oligodendrocytes along with striatonigral degeneration and/or olivopontocerebellar atrophy [10, 52, 67].

GCIs are primarily comprised of aggregated α-synuclein, therefore MSA can be classified as an oligodendroglial α-synucleinopathy, which is a point of distinction compared to neuronal α-synucleinopathies like Parkinson’s disease. Interestingly, work investigating the earliest molecular changes associated with MSA has suggested that oligodendrocyte intracellular accumulation of p25α, a protein associated with myelination, may be altered before α-synuclein aggregation is observed [67]. The aggregation of α-synuclein is thought to lead to a disruption of the role of the oligodendrocyte in the process of neuronal myelination leading to microglial activation and subsequent release of mis-folded α-synuclein by the increasingly dysfunctional oligodendrocytes. Neighboring neurons may uptake this extracellular α-synuclein and it could thereby initiate new aggregation inside the neuronal cell. Additionally, the toxic α-synuclein species may spread to neurons in other synaptically-connected brain regions in a prion-like fashion. The lack of effective oligodendrocyte support for the local neurons, and the neuronal effects of the α-synuclein inclusions, eventually results in axonal dysfunction, neuronal cell death, and a reactive astrogliosis [18].

The cause of MSA is not known, however it is generally believed to be sporadic. Several genomic studies have been performed to shed light on the molecular pathogenesis of the disease. Three SNPs located in the α-synuclein gene (*SCNA*) have been associated with the risk of developing MSA [60]. In an independent study conducted by evaluating 32 SNPs in the *SNCA* gene, one SNP associated with MSA and one haplotype associated with the MSA-C subgroup were noted [2]. Whole genome sequencing analysis identified *COQ2* genetic variants associated with both sporadic and familial MSA [47], and this finding was replicated in other Asian cohorts [41, 79]. In another GWAS, including MSA patients and healthy controls, several SNPs located in different genes (*FBX047*, *ELOVL7*, *EDN1*, and *MAPT*) were found to be potentially associated, but were not significant after multiple test correction [59]. Finally, the presence of an expansion of one allele in *SCA3* (a gene associated with spinocerebellar ataxia) was observed in a patient showing clinical features consistent with MSA-C [48]. Recently, epigenetic modifications, such as DNA methylation changes, have also been identified in neurodegenerative diseases. A recent study reported white matter tissue DNA methylation changes associated with MSA, including changes in *HIP1*, *LMAN2* and *MOBP* [9].

Three different gene expression profiling studies conducted on neuropathologically verified human brain samples have been reported to date. The first study [45] utilized transcriptome profiling by RNA-sequencing of the white and grey matter of the frontal cortex from 6 MSA patients and 6 controls. In the grey matter they detected 5 genes differentially expressed (*HLA-A*, *HLA-B*, *HLA-C*, *TTR* and *LOC389831*). In the white matter they identified 7 genes, including the 3 HLA genes detected in the grey matter. The additional genes were: *HBA1*, *HBA2*, *HBB* and *IL1RL1*. The *SNCA* gene was detected to be upregulated in both comparisons but it was not statistically significant. They also compared the white matter versus the grey matter in patients, detecting a total of 1910 differentially expressed genes. A second study was conducted using the same 12 samples, but using strand-specific RNA-sequencing [46], detecting a total of 123 differentially expressed genes. Most detected genes were lincRNAs or un-annotated transcripts. Some of the genes found in the previous study [45] were confirmed; *HBB*, *IL1RL1*, *TTR* and *LOC389831*. Finally, a study determining the differential expression of circular RNA (circRNA) in the MSA frontal cortex was conducted [14], identifying 5 circRNAs produced by backsplicing of the precursor mRNAs from the *IQCK*, *EFCAB11*, *DTNA*, *MAP4K3*, and *MCTP1* genetic loci. No other RNA sequencing studies have been conducted thus far.

In this study we utilized RNA sequencing to characterize the cerebellar white matter transcriptome from neuropathologically verified MSA cases and controls using two independent sample sets and two different profiling technologies.

## Material and methods

Extended methods are reported in the Supplementary Appendix.

### Human samples

We analyzed two independent cohorts of postmortem cerebellar white matter (CWM) that included both MSA-P and MSA-C subtypes. Cohort 1 (C1) was obtained from the New South Wales (NSW) Brain Banks (Sidney, AU) and from the Brain and Body Donation Program (BBDP; Sun City, AZ) to yield a total of 19 pathologically-proven cases MSA and 10 Healthy Controls (HC) (Table 1a). Specimens were obtained from deep cerebellar white matter lateral to the dentate nucleus. Cohort 2 (C2) was obtained from the Queen Square Brain Bank for Neurological Disorders (London, UK) and included 48 pathologically proven MSA cases and 47 HC (Table 1b). Specimens were obtained from the cerebellar hemisphere.

**Table 1:** Sample characteristics of the different cohorts analyzed. Differences in age and PMI between cases and controls were assessed using t-test or Wilcoxon test, according to the data distribution. Sex distribution was assessed using the Fisher’s Exact test

***A (Cohort 1)***		***MSA (n = 19)***	***HC (n = 19)***	***p***
	***Age***	70.2 ± 7.4	69.6 ± 6.5	*t* = − 0.311; *p* = 0.757
	***PMI***	10.6 ± 10.1	12.0 ± 10.8	*W* = 175, *p* = 0.884
	***Males***	10	10	*p* = 1.000
	***Females***	9	9	
		***MSA-P (n = 5)***	***HC (n*** ***= 19)***	*p*
	***Age***	66.8 ± 5.8	69.6 ± 6.5	*t* = − 1.168; *p* = 0.255
	***PMI***	15.2 ± 5.9	12.0 ± 10.8	*W* = 60.5; *p* = 0.374
	***Males***	3 (60.0)	10 (52.6)	*p* = 1.000
	***Females***	2 (40.0)	9 (47.4)	
		***MSA-C (n = 5)***	***HC (n*** ***= 19)***	*p*
	***Age***	72.2 ± 6.6	69.6 ± 6.5	*t* = 0.366; *p* = 0.718
	***PMI***	19.4 ± 13.2	12.0 ± 10.8	*W* = 66.5; *p* = 0.188
	***Males***	4 (80.0)	10 (52.6)	*p* = 0.356
	***Females***	1 (20.0)	9 (47.4)	
***B (Cohort 2)***^*a*^		***MSA (n = 48)***	***HC (n = 47)***	***p***
	***Age***	64.5 ± 8.0	84.2 ± 9.1	*W* = 159.5; *p* = 5.6E-13
	***PMI***	61.7 ± 24.0	59.9 ± 28.2	*W* = 1172; *p* = 0.746
	***Males (%)***	21 (43.8)	16 (34.0)	*p* = 0.402
	***Females (%)***	27 (56.3)	31 (66.0)	
		***MSA_P (n = 37)***	***HC (n = 47)***	***p***
	***Age***	64.8 ± 8.5	84.2 ± 9.1	*W* = 129.5; *p* = 2.6E-11
	***PMI***	63.2 ± 24.6	59.9 ± 28.2	*W* = 930; *p* = 0.589
	***Males (%)***	14 (37.8)	16 (34.0)	*p* = 0.820
	***Females (%)***	23 (62.1)	31 (66.0)	
		***MSA_C (n = 11)***	***HC (n = 47)***	***p***
	***Age***	63.5 ± 6.4	84.2 ± 9.1	*W* = 30; *p* = 6.0E-06
	***PMI***	56.9 ± 22.2	59.9 ± 28.2	*W* = 242; *p* = 0.751
	***Males (%)***	7 (63.6)	16 (34.0)	*p =* 0.093
	***Females (%)***	4 (36.4)	31 (66.0)	
***C (Cohort 1 - LCM)***^b^		***MSA (n = 6)***	***HC (n = 6)***	***p***
	***Age***	70.0 ± 7.7	72.0 ± 7.0	*t* = −0.469; *p* = 0.650
	***PMI***	16.3 ± 14.4	9.4 ± 11.0	*W* = 26.5; *p* = 0.199
	***Males***	4 (66.7)	2 (33.3)	*p* = 0.547
	***Females***	2 (33.3)	4 (66.7)	

^a^One MSA-P sample was removed after the PCA analysis (Final sample size: MSA = 47; MSA- P = 36; MSA-C = 11; HC = 47)

^b^Two MSA and one HC samples were removed after PCA analysis (Final sample size: MSA = 4; HC = 5)

### RNA extraction and RNA sequencing

For C1, total DNAse-treated RNA was extracted using the Qiagen RNAeasy kit (Qiagen). Quality was assessed by Bioanalyzer (Agilent). Sequencing libraries were prepared with 250 ng of total RNA using Illumina’s Truseq RNA Sample Preparation Kit v2 (Illumina, Inc.) following the manufacturer’s protocol and purifying poly-A mRNAs with poly-T oligos attached to magnetic beads. The final library was sequenced by 50 bp paired-end sequencing on a HiSeq 2500 (Illumina, Inc.). For C2, total DNAse-treated RNA was extracted in TRI Reagent from ~ 5 mg of tissue using Rino Tubes (Next Advance) (TempO-Seq Assay User Guide version 2.0). This technology is based on a proprietary 3′ mRNA capture protocol, using probes for protein-coding mRNAs. Extracted RNA was not rRNA depleted or poly(A)-selected. The final library was sequenced by 50 bp single-end sequencing on a NextSeq 500 (Illumina, Inc.).

### Laser capture microdissection (LCM)

Twelve samples (6 MSA, 6 HC) from C1 were used for laser capture microdissection (LCM) of oligodendrocytes from cerebellar white matter [49]. Oligodendrocytes were stained by using a modified H&E staining protocol adapted from Ordway et al. [49]. A total of 300 oligodendrocytes per sample were captured using Arcturus CapSure Macro LCM Caps (Applied Biosystems) with the following settings: UV speed at 676 um/s and UV current at 2%. RNA was extracted immediately after cell capture using the Arcturus PicoPure RNA Isolation Kit (Applied Biosystems). For library preparation the SMARTer® Stranded Total RNA-Seq Kit - Pico Input (Clontech/Takara) was used, including the integrated removal of rRNAs. Samples were sequenced (2 × 75 bp paired-end run) on the Illumina HiSeq2500.

### Data analysis

Quality controls on FASTQ files were conducted using *MultiQC v0.9* software [26]. The reads were aligned to the human reference genome (GRCh37) using the *STAR* software *v2.5* [23] and summarized as gene-level counts using *featureCounts v1.4.4* [43]. For both datasets (C1 and C2) PCA analysis was used to assess the presence of outliers and to detect any batch effects. Four samples were deemed to be outliers and were removed (detailed below in Results). Gene expression differential analyses between MSA cases and HC were conducted using the R package *DESeq2 v1.14.1* [43], including age, sex (only C2), PMI and sample source (only C1) as covariates. Sex was not included as a covariate for C2 because the sexes were balanced and sample source was not included as a covariate for C1 because the tissue sources were balanced. The *p*-values were corrected for multiple testing using the False Discovery Rate (FDR) method, considering as significant all the genes with adjusted *p*-value (adj *p*) < 0.05. The results from the two cohorts were combined using a meta-analysis approach based on the weighted-Z method [77] as implemented in the R-package *survcomp* [61].

### Cell specific expression

We classified the genes detected in the differential expression analysis using an external database of expression values from different types of cells isolated from mouse cerebral cortex [78]. We computed an enrichment score for each cell type and gene, assigning each gene to a specific cell type according to the relative expression in the other cell types. The method used to generate the enrichment score was described in our previous study, and was used to classify RNA profiling data from human bulk tissue isolated from 7 different brain regions to identify cell specific functional processes in Alzheimer’s Disease [54]. The statistical enrichment of cell specific genes was investigated across DEGs and co-expression modules using a hypergeometric statistic (R function *phyper*).

### Enrichment and functional network analysis

Lists of DEGs were analyzed for Gene Ontology (GO) enrichment using the R-package *anRichmentMethods*, adjusting the *p*-value with the FDR method. The same gene lists were also analyzed using *HumanBase* (https://hb.flatironinstitute.org/gene)*,* constructing tissue-specific functional networks [30].

The enrichment of Alzheimer’s disease genes in MSA was conducted using the data from the Accelerated Medicine Partnership – Alzheimer’s Disease (AMP-AD) portal, available at https://www.synapse.org/#!Synapse:syn14237651. We downloaded the differential expression results from 7 different brain regions from the Mayo, Mount Sinai and ROSMAP cohorts [3, 7, 76]. Specifically, the brain regions included were: temporal cortex (TCX), cerebellum (CBE), dorsolateral pre-frontal cortex (DLPFC), inferior frontal gyrus (IFG), frontal pole (FP), parahippocampal gyrus (PHC), and superior temporal gyrus (STG). The DEGs from these 7 brain regions were used as gene set references for the list of MSA-C genes ranked by log2 FC. The analysis was conducted using the R-package *fgsea* adjusting the *p*-values with the FDR method.

### Weighted correlation network analysis

We conducted Weighted Correlation Network Analysis (WGCNA) in the MSA-C cohorts with the aim of identifying modules of co-expressed genes associated with the disease and enriched for specific biological processes [38]. We computed the co-expression networks using the data from C1 and then we estimated the module preservation in C2, using only MSA-C and HC. The analysis was conducted using the *WGCNA* R-package [38]. Genes for both C1 and C2 with less than 10 average counts were filtered out due to low expression, and data were normalized using the *vst* function of the *DESeq2v1.14.1 *package [40]. The matrix of expression values was adjusted for age, sex, source and PMI using the function *removeBatchEffect* as implemented in the *limma* R-package [57]. Finally, we filtered out the 50% of genes having lower Median Absolute Deviation (MAD). We generated a signed co-expression network for C1 using the function *blockwiseModules*, with the option *mergeCutHeight* = 0.25. Then, we computed the module eigengenes and we investigated their relationship with disease status using a linear model as implemented in the *limma* package. We calculated the module membership and gene-trait significance (MSA-C disease status) with the goal of ranking genes in each co-expression modules. Modules associated with disease status were further investigated using GO enrichment analysis. The enrichment for genes expressed in specific cell types was conducted using as reference gene sets the gene specifically expressed in the 5 cell types from Zhang et al. [78] (see previous section “Cell specific Expression”) and as a test set all of the genes ranked by module memberships for the module associated with disease status. Finally, we checked the module preservation in C2 using the *modulePreservation* function with 1,000 permutations. Relevant coexpression networks were exported and visualized using *Cytoscape v3.7.2* [62]. Results from relevant modules were compared with published data from Darbelli et al. [19], intersecting the lists of genes and conducting an enrichment analysis using the R-package *fgsea*.

## Results

### Quality controls

For C1 (Illumina), we sequenced a total of 470 Million (M) reads (average: 12.4 M; range: 3.8–32.6 M) with a 76.7% average mapping rate. PCA analysis did not show the presence of outliers (Fig. S1). For C2 (TempO-Seq) we sequenced a total of 162 M reads (average: 1.7 M; range: 0.1–3.8 M), with a 90.2% average mapping rate. PCA analysis showed the presence of one outlier in the C2 group and it was removed from all subsequent analyses. The final sample size was: MSA = 47 and HC = 47 (Fig. S2A and Fig. S2B). For the LCM sample (a subsample from C1) we sequenced a total of 353 M reads (average: 29.4 M; range: 23.4–33.3 M) with an average 64.4% mapping rate. We detected the presence of three outliers that were also removed. The final sample size used for the differential analysis from the LCM dataset was: MSA = 4 and HC = 5 (Fig. S3A and Fig. S3B).

### Differential expression results: bulk tissue human samples (MSA, MSA-P and MSA-C vs HC)

Differentially expressed genes (DEGs) were obtained by combining the results from both cohorts using a meta-analysis approach. Details about the specific results for each cohort are reported in Tables S1-S3 and Fig. S4*.* The comparisons of the log2 FC obtained in the differential analyses for the two independent cohorts for MSA, MSA-P and MSA-C were statistically significant (*ρ* range *=* 0.204–0.456; *p* < 2.2E-16)*.* The largest correlation coefficient (*ρ* = 0.456) was obtained for the MSA-C subtype probably due to the larger significance and effect size of the genes detected (Fig. S5).

**Fig. 1:**
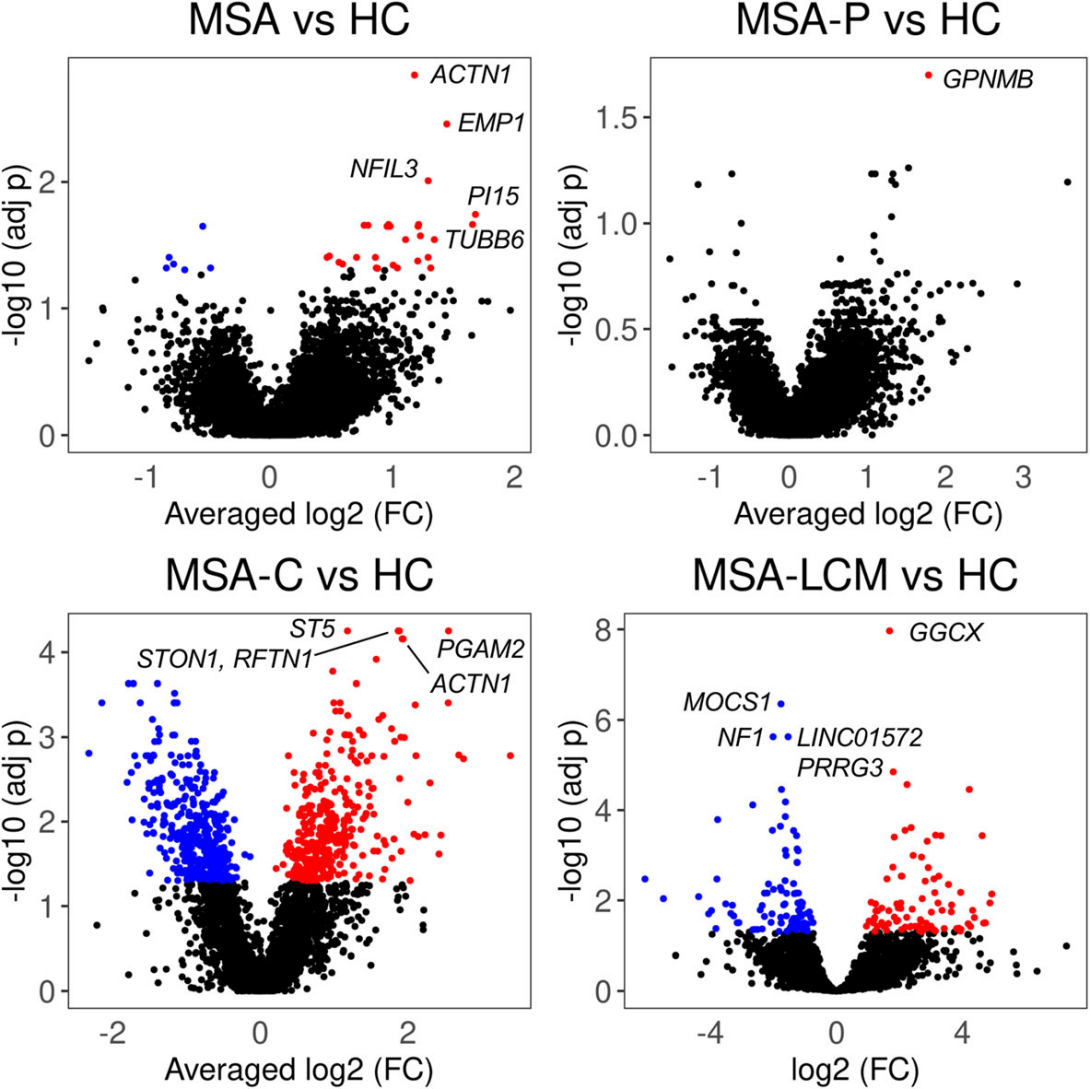
Volcano plots representing the differential expression results after *p*-value combination (excluding LCM dataset). In red and blue upregulated and downregulated genes, respectively. The top 5 genes symbols ranked by *p*-values are shown. **a**: MSA. **b**: MSA-P. **c**: MSA-C. **d**: LCM Oligodendrocytes

**Fig. 2:**
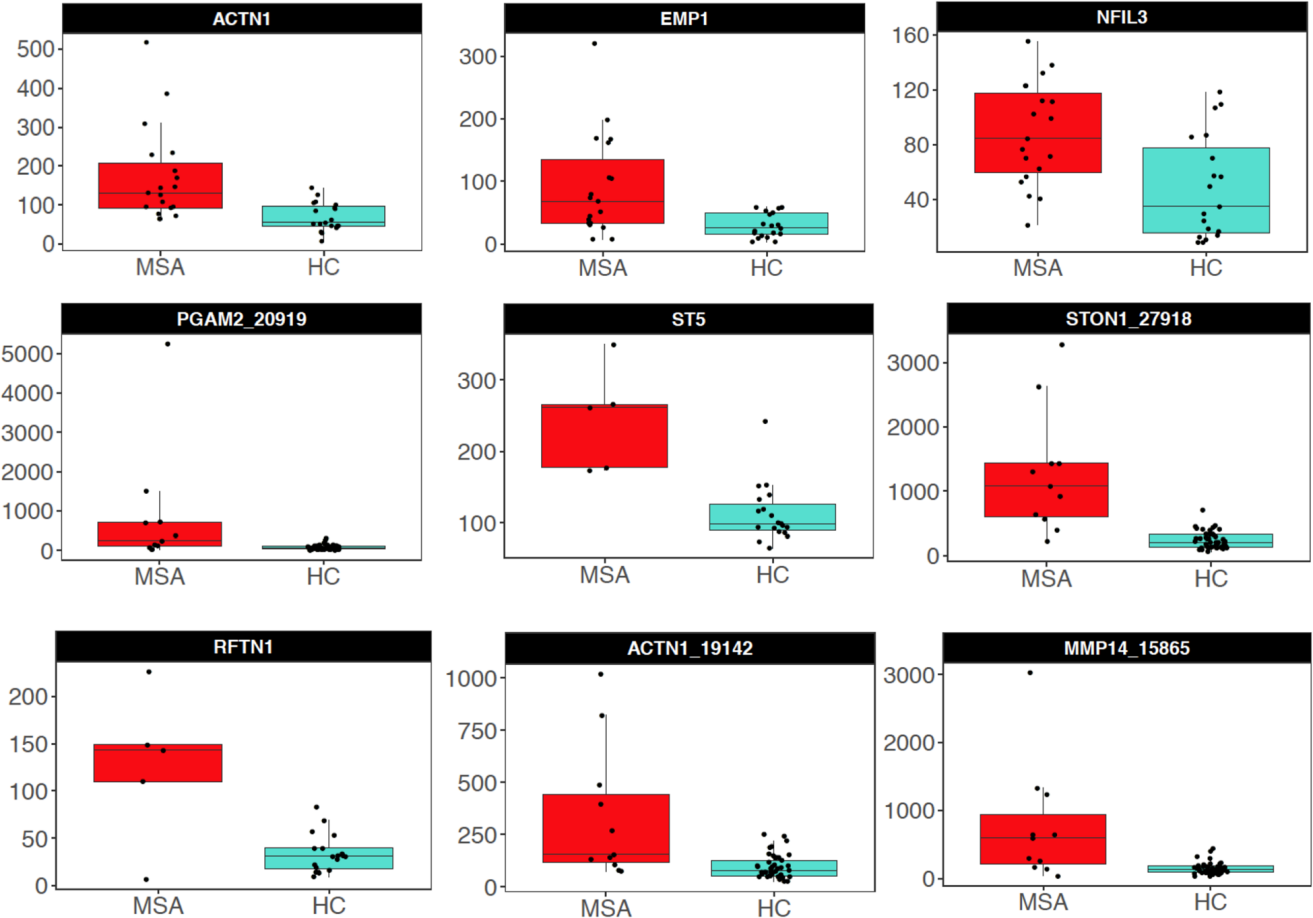
Top differentially expressed genes found in MSA (first row), and MSA-C (second and third row)

After *p*-value combination, we obtained a set of DEGs ranging from 1 (MSA-P) to 747 (MSA-C) depending on the MSA clinical sub-type (Fig. 1a – c). The complete results are reported in Tables S4-S6*.* The top 3 DEGs for MSA in general were *ACTN1*, *EMP1* and *NFIL3* (adj *p* < 0.01; all upregulated). In the MSA-P clinical sub-type we detected only one DEG (*GPNMB*), whereas in MSA-C the top genes were: *PGAM2*, *ST5*, *STON1*, *RFTN1*, *ACTN1* and *MMP14* (adj *p* < 1.0E-04*;* all upregulated) (Table 2; Fig. 2)*.* We explored the differential expression between SND vs HC, and OPCA vs HC, detecting a total of 7 and 58 genes, respectively. *MLPH*, detected in SND, was also detected when analyzing the clinical subtype MSA-P in C2, whereas a total of 47 genes detected in OPCA were also observed in the MSA-C clinical subtype in C2 (Table S7; Fig. S6). Correlation of the log2 FC between the differential analysis for clinical and neuropathological classification criteria were *ρ* = 0.622 (MSA-P/SND) and *ρ* = 0.830 (MSA-C/OPCA) (both *p* < 2.2E-16) (Fig. S7).

**Table 2:** Top genes for the different MSA subtypes after *p*-value combination. Downregulated genes are reported in grey

Group	Genes Info		Differential Expression Cohort 1				Differential Expression Cohort 2				Combined p- values		Averaged log2 FC
	Symbol	Ensembl ID	Base Mean	log2 (FC)	***p***	adj ***p***	Base Mean	log2 (FC)	***p***	adj ***p***	***p***	adj ***p***	
**MSA**	*ACTN1*	ENSG00000072110	120.266	1.309	2.3E-06	1.4E-02	152.163	1.055	1.4E-04	2.0E-01	1.2E-07	1.4E-03	1.182
	*EMP1*	ENSG00000134531	59.778	1.636	7.4E-06	1.6E-02	240.880	1.254	7.3E-04	4.9E-01	5.8E-07	3.5E-03	1.445
	*NFIL3*	ENSG00000165030	68.525	1.019	1.1E-04	4.6E-02	21.457	1.567	5.1E-04	4.1E-01	2.4E-06	9.8E-03	1.293
	*PI15*	ENSG00000137558	13.120	1.086	2.3E-02	3.7E-01	11.510	2.273	9.8E-06	5.1E-02	6.0E-06	1.8E-02	1.679
	*TUBB6*	ENSG00000176014	31.136	1.426	4.5E-06	1.5E-02	0.696	1.885	8.8E-02	1.0E+ 00	9.0E-06	2.2E-02	1.656
	*VIM*	ENSG00000026025	297.903	0.701	7.8E-03	2.5E-01	1841.955	1.240	7.0E-05	1.5E-01	1.1E-05	2.2E-02	0.971
	*COL4A1*	ENSG00000187498	94.979	1.424	1.6E-04	5.6E-02	71.659	1.008	4.3E-03	7.6E-01	1.3E-05	2.2E-02	1.216
	*NFKBIA*	ENSG00000100906	280.164	0.672	9.7E-05	4.1E-02	8.006	0.932	8.9E-03	8.8E-01	1.5E-05	2.2E-02	0.802
	*MAT2A*	ENSG00000168906	766.718	0.641	6.1E-03	2.4E-01	1065.143	0.902	1.6E-04	2.0E-01	1.6E-05	2.2E-02	0.771
	*AEBP1*	ENSG00000106624	238.300	1.638	2.1E-06	1.4E-02	14.682	0.328	4.5E-01	1.0E+ 00	1.9E-05	2.2E-02	0.983
**MSA-P**	*GPNMB*	ENSG00000136235	87.507	1.798	1.5E-03	3.0E-01	34.116	1.773	2.2E-05	7.7E-02	1.7E-06	2.0E-02	1.785
**MSA-C**	*PGAM2*	ENSG00000164708	64.618	1.906	4.7E-04	3.6E-02	230.022	3.193	3.5E-08	4.8E-05	2.7E-08	5.6E-05	2.549
	*ST5*	ENSG00000166444	140.163	1.216	6.0E-07	7.8E-04	79.401	1.146	8.8E-05	6.7E-03	4.8E-08	5.6E-05	1.181
	*STON1*	ENSG00000243244	59.570	1.560	1.1E-03	5.7E-02	433.908	2.177	1.0E-08	2.7E-05	2.3E-08	5.6E-05	1.868
	*RFTN1*	ENSG00000131378	53.043	2.423	7.5E-07	8.6E-04	66.900	1.345	6.2E-05	6.0E-03	4.5E-08	5.6E-05	1.884
	*ACTN1*	ENSG00000072110	101.733	1.939	2.9E-05	9.3E-03	141.214	1.905	5.7E-06	1.5E-03	8.6E-08	7.0E-05	1.922
	*MMP14*	ENSG00000157227	68.018	1.740	1.5E-04	2.0E-02	270.406	2.128	1.2E-06	5.8E-04	9.0E-08	7.0E-05	1.934
	*ITGB4*	ENSG00000132470	425.361	1.491	1.2E-04	1.8E-02	109.863	1.650	6.0E-06	1.5E-03	1.8E-07	1.2E-04	1.570
	*MAPK4*	ENSG00000141639	274.832	1.561	3.6E-09	6.0E-05	53.534	0.402	3.6E-01	6.5E-01	2.9E-07	1.7E-04	0.981
	*OMG*	ENSG00000126861	431.926	−1.384	2.3E-03	8.0E-02	56.937	−2.185	2.5E-06	9.7E-04	6.0E-07	2.3E-04	−1.785
	*FAM107A*	ENSG00000168309	2370.587	0.848	9.9E-03	1.6E-01	2751.910	1.754	3.5E-07	2.7E-04	4.6E-07	2.3E-04	1.301

**Fig. 3:**
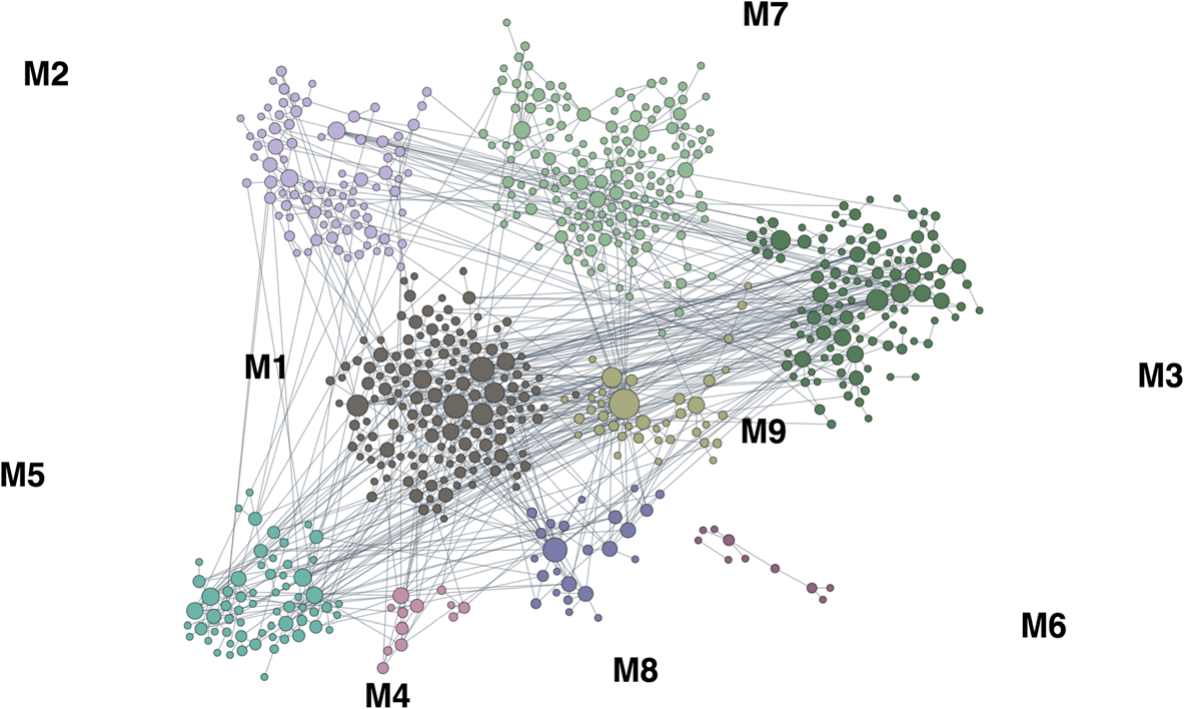
Results of the functional network analysis on MSA-C DEGs. Module 1 was enriched for amyloid-β metabolism (*q* = 5.3E-05) including key AD genes as: *APP*, *PSEN1*, *CLU*, *ROCK2* and *DYRK1*

We explored the functional significance of the DEGs by applying a functional network analysis specific for the cerebellum and a GO enrichment analysis. Using the 35 MSA DEGs (Table S4) we detected a small network with 2 modules enriched for “cell-cell adhesion” (*SELL* and *BCL6* genes) and “angiogenesis” (*COL4A1* and *COL4A2* genes) (both *q* < 0.01) (Fig. S8). The GO analysis yielded significant enrichment of the Biological Process “collagen-activated signaling pathway” (adj *p* = 0.030; genes: *COL4A1*, *COL4A2*, *ITGA11).* Using all of the 747 MSA-C DEGs (Table S6) we detected a large network including 9 different modules (Fig. 3). We observed the highest enrichment significance in module 1 (M1) which was amyloid-β metabolism (top GO process: *q* = 5.3E-05, Table S8), including the Alzheimer’s disease (AD) relevant genes: *APP*, *PSEN1*, *CLU, ROCK2* and *DYRK1*. The central role of *APP* was confirmed by a separate protein-protein interaction analysis showing this gene as the most important hub in a network that included 30% of the DEGs generated using *WEBGESTALT* [75] (Fig. S9)*.* The second highest significance was reached in module 2 (M2) for respiratory chain complex assembly (top GO process: *q* = 8.2E-03) (Table 3, Table S8). With the GO analysis we detected 625 significant functional classes, mostly related to cellular and cytoplasmic components, neuro and gliogenesis (Fig. S10).

**Table 3:** Top results of the functional module discovery analysis using the DEGs identified in MSA-C

CLUSTER (Genes)	TERMNAME	GOID	Q VALUE	GENE COUNT	TERMGENES
**M1 (152)**	amyloid-beta formation	GO:0034205	5.3E-05	6	*ROCK2,DYRK1A,CLU,PSEN1,EFNA1,APP*
	amyloid precursor protein catabolic process	GO:0042987	1.0E-04	6	*ROCK2,DYRK1A,CLU,PSEN1,EFNA1,APP*
	amyloid-beta metabolic process	GO:0050435	1.0E-04	6	*ROCK2,DYRK1A,CLU,PSEN1,EFNA1,APP*
	amyloid precursor protein metabolic process	GO:0042982	2.8E-04	6	*ROCK2,DYRK1A,CLU,PSEN1,EFNA1,APP*
	amyloid fibril formation	GO:1990000	1.0E-03	4	*CLU,GSN,APP,PSEN1*
**M2 (85)**	NADH dehydrogenase complex assembly	GO:0010257	8.2E-03	4	*NDUFA1,NDUFS5,NDUFB3,NDUFB5*
	mitochondrial respiratory chain complex I assembly	GO:0032981	8.2E-03	4	*NDUFA1,NDUFS5,NDUFB3,NDUFB5*
	mitochondrion organization	GO:0007005	1.6E-02	7	*SLC25A5,NDUFB3,NDUFB5,PARP1,PSMD10,NDUFS5,NDUFA1*
	mitochondrial respiratory chain complex assembly	GO:0033108	1.8E-02	4	*NDUFA1,NDUFS5,NDUFB3,NDUFB5*
	negative regulation of centriole replication	GO:0046600	2.0E-02	2	*RBM14,CHMP2A*
**M3 (117)**	regulation of cellular protein localization	GO:1903827	1.1E-02	9	*EZR,IWS1,RDX,GPSM2,NUMB,PICALM,RTN4,BAG3,UHMK1*
	regulation of organelle assembly	GO:1902115	1.4E-02	6	*STAG1,EZR,CCP110,GPSM2,RDX,CHMP5*
	sulfur compound biosynthetic process	GO:0044272	1.7E-02	4	*MTRR,GCLC,MAT2A,PAPSS1*
	membrane docking	GO:0022406	1.8E-02	3	*PDZD8,EZR,ATG14*
	regulation of protein export from nucleus	GO:0046825	2.3E-02	3	*BAG3,IWS1,UHMK1*
**M4 (12)**	negative regulation of multi-organism process	GO:0043901	1.3E-02	3	*IFITM3,TIMP1,ANXA2*
	regulation of multi-organism process	GO:0043900	2.8E-02	3	*IFITM3,TIMP1,ANXA2*
	negative regulation of protein catabolic process	GO:0042177	3.3E-02	2	*TIMP1,ANXA2*
	skeletal system development	GO:0001501	3.9E-02	2	*CD44,ANXA2*
	negative regulation of endopeptidase activity	GO:0010951	4.0E-02	2	*CD44,TIMP1*
**M5 (63)**	integrin-mediated signaling pathway	GO:0007229	2.2E-02	3	*FLNA,LAMA5,ZYX*
	positive regulation of cell development	GO:0010720	2.8E-02	4	*NSMF,FLNA,ARHGEF2,PLXNB2*
	actin cytoskeleton organization	GO:0030036	4.0E-02	5	*FSCN1,RHOG,FLNA,ZYX,ARHGEF2*
	actin filament organization	GO:0007015	4.1E-02	4	*FSCN1,FLNA,ARHGEF2,ZYX*
	supramolecular fiber organization	GO:0097435	4.1E-02	5	*FSCN1,FLNA,ZYX,B4GALT7,ARHGEF2*
**M6 (10)**	calcium ion transport	GO:0006816	4.0E-02	2	*CDH23,PRKG1*
	divalent metal ion transport	GO:0070838	4.1E-02	2	*CDH23,PRKG1*
	divalent inorganic cation transport	GO:0072511	4.1E-02	2	*CDH23,PRKG1*
**M7 (164)**	synapse organization	GO:0050808	4.4E-02	3	*CNTN2,NLGN3,SLITRK1*
**M8 (24)**	renal system development	GO:0072001	4.7E-02	2	*COL4A1,ITGB4*
	urogenital system development	GO:0001655	4.9E-02	2	*COL4A1,ITGB4*

### Differential Expression analysis: bulk tissue human samples (MSA-C vs MSA-P, OPCA vs SND)

We ran the comparison MSA-C vs MSA-P in C1 and C2, obtaining 1 DEG in C1 (Table S9). After *p*-value combination we did not detect any remaining significant genes after multiple test correction. The top 10 genes ranked by *p*-value are reported in Table S10. Finally, in the comparison of OPCA vs SND in C2 we obtained 156 DEGs (Table S11 and Figure S11). The gene detected in MSA-C vs MSA-P (*PDZRN4*) was not detected in the OPCA vs SND analysis. The GO enrichment for these DEGs is shown in Table S12, with top processes being “nervous system development” and “neurogenesis”.

### Enrichment of AD genes in MSA-C

After we observed the presence of the AD-related process (amyloid-β metabolism) and AD-related genes in MSA-C, we tested the enrichment of AD genes in MSA-C. We used data from AMP-AD, running an enrichment analysis by brain region using as reference sets the DEGs from each brain region. The results showed a significant enrichment of TCX (adj *p* = 7.4E-05) and PHG (adj *p* = 2.0E-02) AD DEGs among upregulated MSA-C genes (Fig. S12) which were also confirmed when we used more conservative cutoffs to select AD genes (adj *p* < 0.01, < 0.001, and < 0.0001) (Table S9A). As further validation, we used the less variable genes between AD and non-demented controls (ND) (adj *p* > 0.950). As expected, we did not observe any significant enrichment of TCX or PHG AD DEGs genes (Table S13). We compared the DEGs detected in MSA-C, with the DEGs detected in TCX and PHG, only selecting genes having the same log2 FC direction, considering the comparison: affected vs non-affected. We detected 243 genes in TCX, 166 in PHG and 126 common between both regions TCX, PHG and MSA-C (Table S14).

### Differential expression in LCM oligodendrocytes

We detected a total of 187 differentially expressed genes in oligodendrocytes (90 upregulated and 97 downregulated) (Fig. 1d). Details for the complete list of genes are reported in Table S15. The top 4 significant genes (adj *p* < 1.0E-05) were: *GGCX*, *MOCS1*, *NF1* and *LINC01572* (Table 4). Using functional module discovery analysis, we detected a network including 4 modules (72 genes in total) enriched for telomere maintenance (M1: *q* = 1.9E-03; genes: *PTGES3* and *WRAP53*) and ncRNA processing (M2: *q* = 0.0025; genes: *DIMT1*, *INTS8*, and *MTREX*). Modules 3 and 4 are weakly enriched for immune processes and cell growth (*q* < 0.05) (Fig. S13). Using the GO analysis in the complete gene list, we detected a significant enrichment in the myelination process mostly due to downregulated genes (Fig. S14).

**Table 4:** Top genes differentially expressed in oligodendrocytes in MSA vs HC

Genes	Ensembl Gene ID	Biotype	Base Mean	log2 FC	Stat	***p***	adj ***p***
*GGCX*	ENSG00000115486	Protein Coding	803.4	1.691	7.072	1.5E-12	1.1E-08
*MOCS1*	ENSG00000124615	Protein Coding	405.2	−1.759	−6.433	1.2E-10	4.4E-07
*NF1*	ENSG00000196712	Protein Coding	775.5	−1.532	−6.089	1.1E-09	2.4E-06
*LINC01572*	ENSG00000261008	lincRNA	207.2	−2.010	−6.063	1.3E-09	2.4E-06
*PRRG3*	ENSG00000130032	Protein Coding	269.3	1.808	5.731	1.0E-08	1.4E-05
*HMBOX1*	ENSG00000147421	Protein Coding	124.9	2.247	5.588	2.3E-08	2.7E-05
*PLP1*	ENSG00000123560	Protein Coding	383.7	−1.744	−5.494	3.9E-08	3.5E-05
*–*	ENSG00000249906	antisense	29.0	4.224	5.495	3.9E-08	3.5E-05
*PPP1CA*	ENSG00000172531	Protein Coding	458.2	−1.617	−5.359	8.4E-08	6.6E-05
*C8orf88*	ENSG00000253250	Protein Coding	46.1	−2.657	−5.313	1.1E-07	7.6E-05

### Bioinformatic-based cell specific expression profiling

**Fig. 4:**
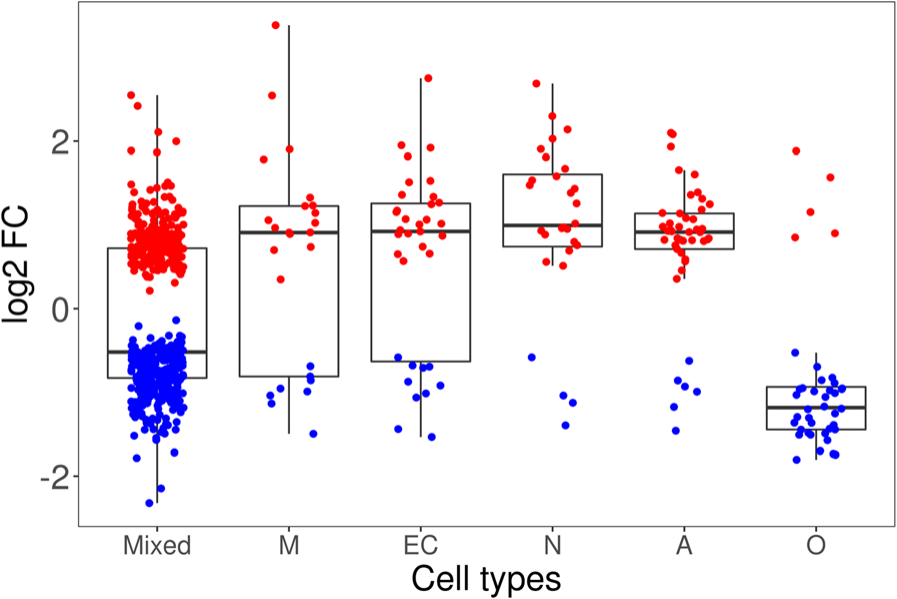
DEGs log2 FC distribution across the cell-specific genes classes. Upregulated and downregulated genes in MSA-C are in red and blue, respectively

**Fig. 5:**
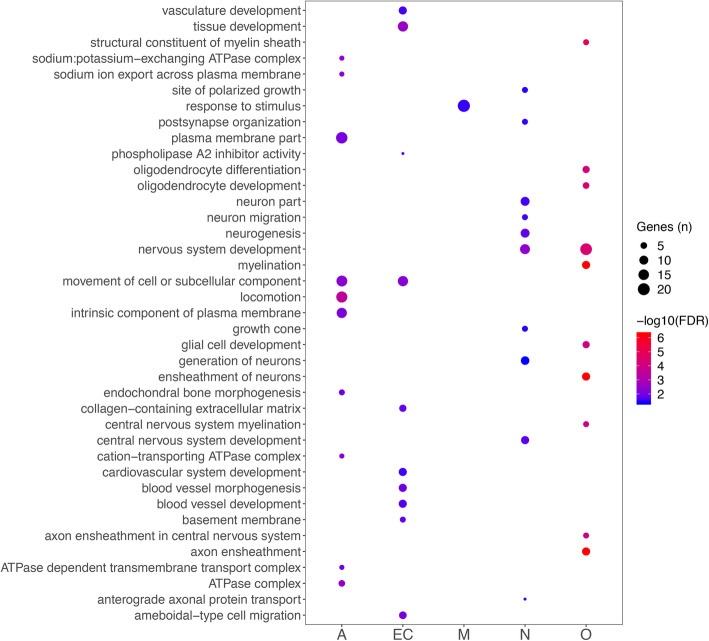
Dot plot reporting the top 10 GO functional classes enriched in each cell-specific gene class

We classified the DEGs obtained in the MSA-C group according to their expression in five brain cell types [54, 78]. We selected only the MSA-C results because the large number of DEGs makes it possible to identify robust cell-specific upregulation/downregulation trends. Most of the DEGs were not cell specific (“mixed”: 74.7% of the total DEGs), whereas the remaining genes were: astrocyte (6.6%), oligodendrocyte (5.8%), endothelial cell (5.1%), neuron (4.1%) and microglia (3.7%) specific. We found a significant overrepresentation of astrocyte and oligodendrocyte genes (both: adj *p* = 2.9E-04). (Fig. S15). We observed a strong downregulation of oligodendrocyte genes and upregulation of microglia, neuron and astrocyte genes (Fig. 4a). To investigate if these patterns are disease specific, we compared the log2 FC of genes differentially expressed (adj *p* < 0.05) with those non-differentially expressed (adj *p* ≥ 0.05) for each cell type. We observed the highest significance for oligodendrocyte (downregulated in MSA) and neuronal genes (upregulated in MSA) (*p* < 0.001). Similar results were obtained when we relaxed the gene inclusion cutoff to adj *p* < 0.10 (Fig. S16). We conducted GO enrichment analysis on the cell-specific DEGs. The highest significance was reached for oligodendrocyte genes, enriched for myelination and oligodendrocyte development processes. Astrocytes were enriched for transport of ion across the membrane, plasma membrane components, and ATPase complex (*FDR* < 0.01). Endothelial cell genes were enriched for cell migration and angiogenesis. Neuronal genes were enriched for neurogenesis and post-synapse organization (Fig. 5; Table S16).

### WGCNA analysis

**Fig. 6:**
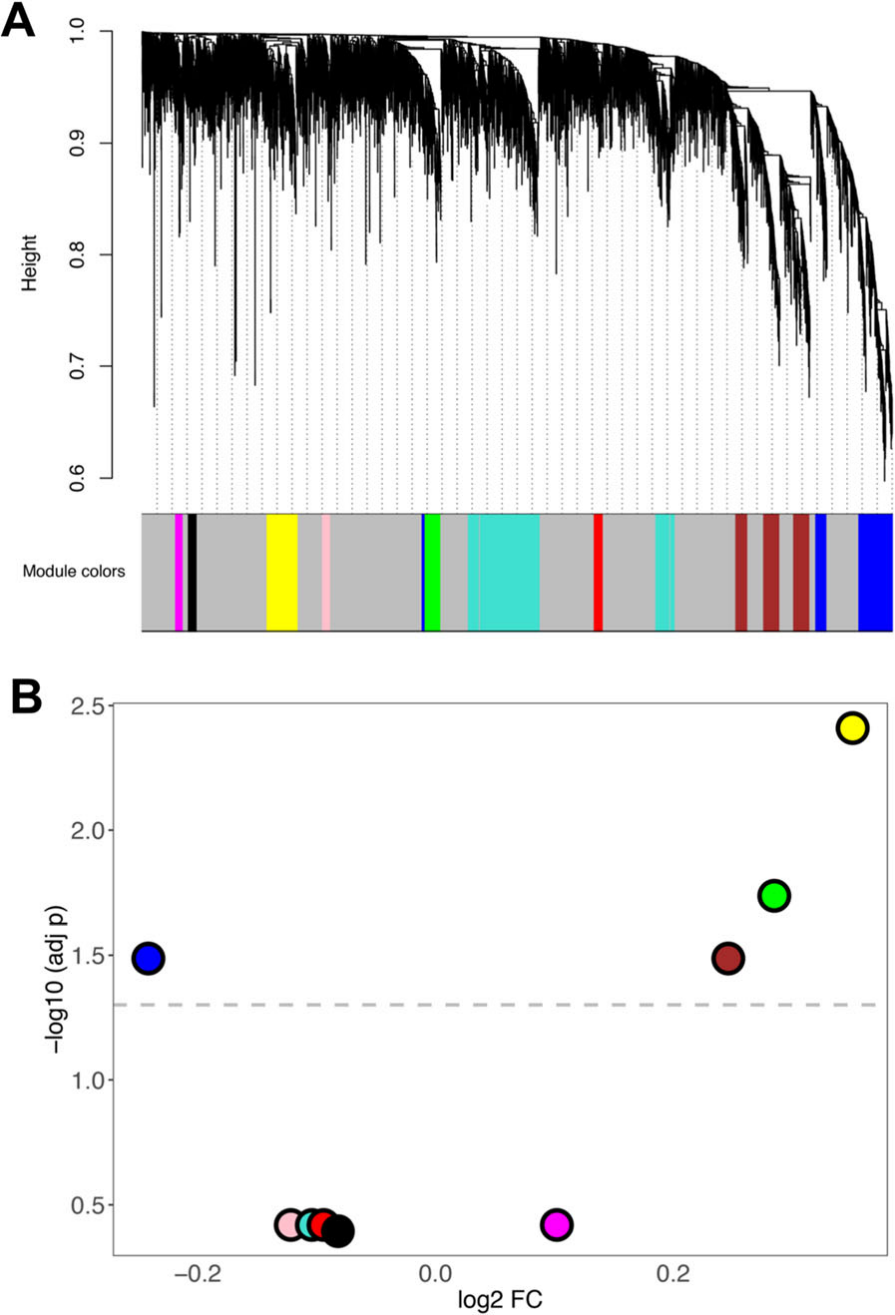
WGCNA analysis. **a** Cluster dendrogram showing the 9 coexpression modules detected in MSA-C (C1). **b** Volcano plot representing the results of the differential expression of the eigengene modules between MSA-C vs HC

**Fig. 7:**
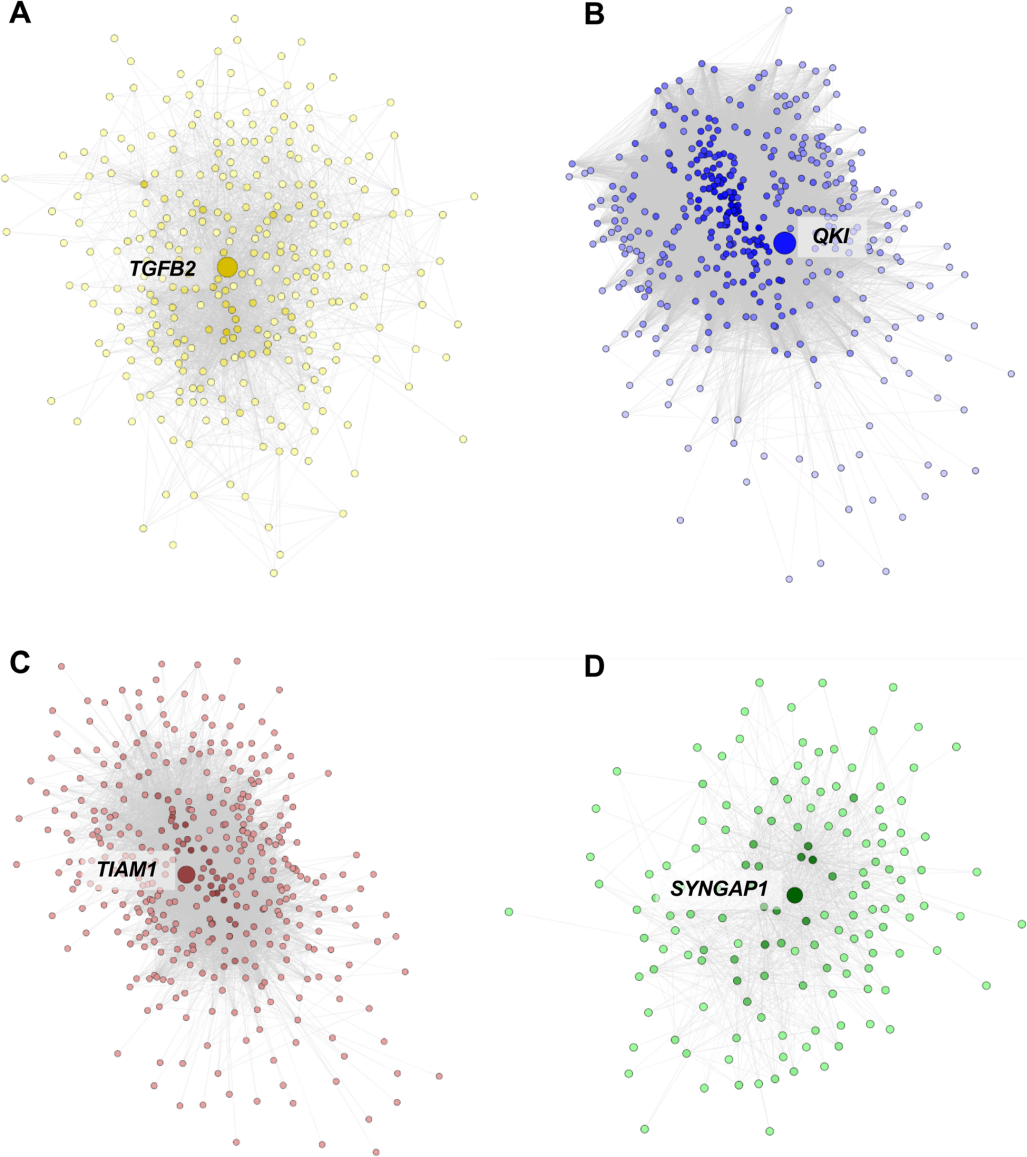
Coexpression network generated from the significant coexpression modules visualized with Cytoscape. The hub genes are the larger nodes. The color intensity of each node is proportional to the number of connections. **a** Network for the yellow module, upregulated in MSA-C. We represented edges with weight ≥ 0.05. **b** Network for the blue module, downregulated in MSA-C. We exported edges with weight ≥ 0.20. **c** Network for the brown module, upregulated in MSA-C. We exported edges with weight ≥ 0.20. **d** Network for the green module, upregulated in MSA-C. We exported edges with weight ≥ 0.01

Considering the large number of DEGs for the MSA-C subtype, we further investigated this group using WGCNA analysis. We computed a coexpression network using the data from C1 and validated the results in C2 by means of the module preservation analysis. After gene filtering (see Methods), a network was generated using the 7650 genes with larger MAD using “9” as threshold power (Fig. S17). We obtained nine co-expression modules in total including 2675 genes (35.0% of the genes), whereas the remaining were not significantly co-expressed and then were included in the grey module (Fig. 6a). The number of the genes in each module ranged from 78 (magenta) to 917 (turquoise). In Fig. S18 we show the heatmap and the dendrogram indicating the correlation between modules. A total of 4 modules (yellow, green, brown and blue) were associated with disease status, all showing an increase in MSA-C with the exception of the blue module (Fig. 6b). The number of genes in these 4 modules ranged from 160 (green) to 485 (blue). Two of the significant modules (brown and green) were highly correlated with each other (Fig. S18). We computed the module membership (correlation of each gene with the module eigengenes), and the gene-trait significance (correlation with disease status). As expected, the gene-trait significance was highly correlated with the log2 FC (*r* = 0.846; *p* < 2.2E-16). We represented the correlation of the module membership with gene-trait significance in the scatterplots in Fig. S19 and Fig. S20. As expected, we detected a significant positive correlation for the 4 modules associated with MSA (yellow, green, brown, and blue in the Figures) but not for the others (not shown). The genes for these 4 significant modules are reported in Tables S17 ranked by module membership *p*-value. The most important hubs for the 4 modules were: *TGFB2* (yellow), *SYNGAP1* (green), *TIAM1* (brown) and *QKI* (blue). These networks are represented in Fig. 7.

We conducted GO enrichment analysis using the gene modules as input, and observed the most significant and specific enrichment in the brown and blue modules (Fig. S21 and Table S18). The yellow module (upregulated) showed a heterogeneous enrichment, including immune response but also tissue and organ development and response to stress. The green module (upregulated) was enriched for membrane proteins, ribosome and translation. The brown module (upregulated) was enriched for synaptic functional classes (top class: FDR = 1.2E-33), and the blue module (downregulated) was enriched for myelination and oligodendrocyte classes (top class: FDR = 3.1E-09).

We explored the enrichment for specific brain cell types gene expression using the data from Zhang et al. [54, 78]. Accordingly with the GO enrichment we observed, we found a significant enrichment of astrocyte (adj *p* = 3.3E-19) and endothelial genes (adj *p* = 2.8E-04) in the yellow module (upregulated, enriched for immunity and organ development), and a significant enrichment of neuronal genes (adj *p* = 2.5E-60) in the brown module (upregulated, enriched for synaptic processes). Furthermore, we detected a significant enrichment of oligodendrocyte genes (adj *p* = 7.7E-33) in the blue module (downregulated, enriched for myelination) (Fig. S22).

We validated the results conducting module preservation analysis, using C2 as the test sample. We observed strong evidence of preservation for the blue (myelination) and brown (synapse) modules, and moderate evidence of preservation in the green module (translation). No evidence of preservation was detected for the yellow module (Fig. S23).

Finally, we conducted a cross-comparison between the genes in the blue module (*QKI*) with the data from Darbelli et al. [19], containing a list of 1899 differentially expressed genes after comparison of oligodendrocyte-specific QKI-deficient mice vs controls. After intersecting the two lists we detected 40.8% (*n* = 198) of the 485 genes in the blue module shared with the QKI-deficient DEGs (Table S19). Additionally, 93.9% of the shared genes showed a concordant log2 FC (downregulation), with a significant positive correlation between the two datasets (*ρ* = 0.311; *p* = 8.0E-06) (Figure S24A). We estimated the enrichment of *QKI* module genes in the QKI-deficient list by GSEA, detecting a significant enrichment across the downregulated genes (Enrichment score = − 0.627; *p* = 1.07E-05) (Figure S24B).

## Discussion

### Overview

We conducted a genome-wide expression profiling study using cerebellar white matter (CWM) homogenates and LCM purified oligodendrocytes from MSA patients and healthy controls (HC). Two independent cohorts were analyzed using different expression profiling approaches and the differentially expressed genes were prioritized using meta-analysis techniques. WGCNA was applied to find clusters of genes functionally related and associated with the disease. This is the largest RNA profiling study conducted on post-mortem brain samples from MSA patients to date.

### Differential dysregulation in MSA subtypes demonstrates more CWM transcriptional changes in MSA-C than in MSA-P

After *p*-value combination, we obtained the largest number of DEGs in the MSA-C subgroup comparison (*n* = 747). Only one gene was noted to be differentially expressed in the MSA-P sub-type analysis. Of note, the majority of the MSA-P patients had demonstrable GCIs in the CWM and 35 DEGs were identified when the MSA cohort was utilized as a single group in a case/control analysis (MSA-C plus MSA-P vs. HC). Of note, the ratio of MSA-P:MSA-C was 2.6:1 therefore the decreased number of DEGs noted in the combined MSA analysis is likely due to the higher number of MSA-P patients in our study. These results agree with the larger involvement of CWM alterations in MSA-C compared to MSA-P during the early stages of the disease [21, 58]. It is possible that due to the early involvement of CWM in MSA-C there is a longer time for the disease-related transcriptional changes to develop in the CWM in these patients. Roncevic et al. [58] found more cerebellar and pontine involvement in MSA-C compared to MSA-P. Dash et al. [21] used voxel-based morphometry (VBM) and diffusion tensor imaging (DTI) to assess the WM and GM changes in the two MSA subtypes and healthy controls. In comparison to controls, MSA-C showed widespread WM changes in supratentorial and infratentorial regions, whereas MSA-P only showed the involvement of association tracts. Their comparison between MSA-C and MSA-P confirmed a greater prevalence of cerebellar changes in MSA-C patients.

### Oligodendrocyte genes are downregulated and enriched for myelination processes

Results from multiple analyses in our study (bulk tissue and LCM RNA sequencing) converge on strong evidence of the dysregulation of oligodendrocyte genes in MSA-C. WGCNA analysis conducted using bulk tissue expression data showed the presence of a coexpression module (blue, *n* = 485 genes) negatively associated with disease status in MSA-C, enriched for myelination processes, and showing a significant enrichment of oligodendrocyte expressed genes in comparison to the other modules. Additionally, this module showed a strong preservation in the independent C2 dataset. The top hub gene in this blue coexpression network was *QKI.* This gene (downregulated in MSA-C) encodes for an RNA-binding protein involved in myelination and oligodendrocyte differentiation [1]. Darbelli et al. [19] conducted a transcriptomic analysis of oligodendrocyte-specific QKI conditional knock-out (KO) mouse brain and found approximately 1800 genes differentially expressed. The underlying functional annotation of these genes was enriched for axon ensheathment and myelination. Moreover, they detected 810 alternatively spliced genes in the conditional KO animals. These results suggest a potential key role of *QKI* as a regulator of RNA metabolism and alternative splicing in oligodendrocytes. The comparison of the genes included in the *QKI* (blue) module with the list from Darbelli et al. [19] showed a significant statistical enrichment across downregulated genes, strengthening our results.

Interestingly, key myelin genes, including *MBP*, *MAG*, *MOBP*, and *PLP1* were all included in the *QKI* module, and also downregulated in the QKI-KO mice. The study by Bettencourt et al. [9] reported MSA-associated DNA methylation changes in *MOBP*, suggesting that the observed downregulation of this gene in MSA might be regulated by changes in DNA methylation levels. For these reasons, we propose that *QKI* is an important candidate gene for MSA. This gene encodes an RNA-binding protein that regulates pre-mRNA splicing, export of mRNAs from the nucleus, protein translation, and mRNA stability. QKI is also a known regulator of oligodendrocyte differentiation and myelination [20, 39, 64], but not of cell death [63]. In a recent study Zhou et al. [82] used a conditional QKI-KO mouse showing that the turnover of the structural lipid components of the mature myelin is controlled by QKI via coactivation of peroxisome proliferator-activated receptor β–retinoid X receptor α (PPARβ-RXRα) complex. Interestingly, they also found PPARβ and RXR agonists (KD3010, bexarotene) alleviate QKI deficiency–induced demyelination. These findings might open new possibilities about exploring potential MSA treatments with the goal of reducing myelin dysfunction via the QKI biological pathway.

As mentioned in the Introduction, the relocalization of p25α from the myelin sheath to the oligodendrocyte soma is one of the earliest molecular events that may trigger α-synuclein aggregation. This process may also slow oligodendrocyte precursor cell maturation by the α-synuclein mediated downregulation of myelin-gene regulatory factor and myelin basic protein [44]. Interestingly, the gene coding for p25α (*TPPP*), which is expressed in oligodendrocytes, was detected to be significantly downregulated in MSA-C patients in our study (adj *p* < 0.05). Additionally, *SNCA* was significantly downregulated after *p*-value combination in MSA-C (adj *p* < 0.05). The same result was found in another study [37], but not confirmed in other work [35, 53]. Other studies based on oligodendrocyte isolation and qPCA analysis described a basal expression and a trend of an increased expression in MSA patients [5, 22].

In the LCM study, relevant genes associated with the myelination process were *NF1*, *PLP1* and *ERMN*. *NF1* (Neurofibromin 1) was downregulated in MSA and it encodes for a protein specialized in the formation of myelin sheaths. Mutations in this gene cause Neurofibromatosis type 1, which is characterized by the growth of tumors along nerves in various parts of the body including the brain. *PLP1* (Proteolipid Protein 1) is specifically expressed in oligodendrocytes and it was also downregulated in MSA-C patients in our sample. The protein product is a predominant component of myelin, and it also has a role in the maintenance of the myelin sheath as well as in oligodendrocyte development and axonal maintenance. Groh et al. [31] showed that mice with a loss of function *PLP1* mutation exhibit neuroinflammation that leads to axonal degeneration and neuronal cell loss. Finally, *ERMN* (Ermin), downregulated in MSA-C, is involved in myelinogenesis and in maintenance and stability of the myelin sheath.

It is worth mentioning other genes highly differentially expressed in the LCM study even if not directly functionally associated with myelination: *GGCX*, and *MOCS*. *GGCX* (Gamma-Glutamyl Carboxylase) was upregulated in MSA patients, and it is essential for activating vitamin K-dependent proteins [69]. Mutations in this gene cause the “GGCX Syndrome” (OMIM: 137167). It has been observed in vitro that Vitamin K delays α-synuclein fibrillization through its interaction at specific sites at the N-terminus of α-synuclein [65]. *MOCS1* (Molybdenum Cofactor Synthesis 1) was downregulated and it is involved in the biological activation of molybdenum. Mutations in *MOCS1* causes molybdenum cofactor deficiency which is characterized by neurodegeneration and seizures [6].

The results of the LCM study were not from two independent cohorts, as in the case of the bulk tissue results. However, since the same samples were also characterized using bulk tissue, we detected a high log2 FC concordance rate (80%) between the two experiments for oligodendrocyte genes in the top 50% distribution of the FDR values. Additional details of this analysis are reported in our previous study [54].

### Neuron cell-specific genes are upregulated in MSA CWM and are enriched for biological pathways related to synaptic processes

Two different analytical approaches suggested significant upregulation of neuronal cell-specific genes in MSA-C and these genes were enriched for biological roles in synaptic and neurogenesis processes. When we classified the DEGs from MSA-C according to our cell-specific gene analysis approach, we detected an upregulation of neuronal genes and an enrichment for synaptic and neuronal processes. Using WGCNA analysis we detected a module of 451 co-expressed genes (brown) significantly upregulated in MSA tissue and enriched for synaptic processes. The genes in this “brown” module demonstrated a higher prevalence of neuronal-specific genes in comparison to the other significant modules. As was the case with the blue module (discussed above), the brown module showed strong model preservation in our independent C2 dataset. The hub gene in the brown module co-expression network was *TIAM1* (T Cell Lymphoma Invasion and Metastasis 1). This gene (upregulated in MSA-C CWM) encodes a RAC1-specific guanine nucleotide exchange factor that is involved in the control of excitatory synapse development [72]. Interestingly, the green module (significantly upregulated in MSA-C CWM) was correlated with the brown module and showed an enrichment in protein transport and translation. The hub gene in this module was *SYNGAP1* (Synaptic Ras GTPase Activating Protein 1, upregulated in MSA-C) which, like *TIAM1*, is also involved in synaptogenesis [8, 17]. The upregulation of neuron-specific genes and the enrichment for synaptogenesis is surprising in the context of a neurodegenerative disease like MSA. Monomeric α-synuclein is normally located in the presynaptic nerve terminals and is involved in synaptogenesis [80, 81]. Perhaps, the enrichment of the synaptogenesis process in MSA-C CWM in our study might be a consequence of an abnormal accumulation of α-synuclein in the synapse of MSA patients. This elevated synaptic accumulation was previously described to precede the re-localization of α-synuclein from neurons to oligodendrocytes and may represent one of the earliest and ongoing molecular events associated with the disease [68]. Alternatively, this upregulation of synaptogenesis in the context of neurodegeneration in the MSA-C brain may represent a transcriptional attempt by the remaining neurons to compensate for the overall synaptic losses within the CWM.

### The importance of neuroinflammation in MSA-C

The combined relocalization of p25α and the ectopic presence of α-synuclein in oligodendrocytes are thought to trigger the formation of α-synuclein and p25α inclusions [50, 66]. These inclusions and resulting oligodendrocyte dysfunction, activate microglia and astrocytes contributing to the neurodegenerative process through neuroinflammation [27]. These phenomena may explain our finding of the upregulated yellow module (314 genes). This module includes a large prevalence of astrocyte and microglia genes compared to the other significant modules and it is enriched for inflammatory and tissue/organ developmental processes. We found that astrocyte and endothelial specific genes were significantly upregulated in the DEGs from bulk tissue. The top hub gene in the yellow module was *TGFB2* (Transforming Growth Factor Beta 2). This gene encodes a secreted ligand of the TGF-beta (transforming growth factor-beta) superfamily of proteins that are involved in the recruitment and activation of SMAD family transcription factors. Interestingly, the levels of TGFβ-2 were previously found to be increased in the neocortex of AD and dementia with Lewy bodies and were positively correlated with neuropathological markers of disease severity [16]. This finding may suggest that TGF-beta is a key regulator of the inflammatory processes that may be more generalizable to neurodegenerative diseases regardless of the underlying causes and resulting neuropathologies. In the yellow module we found also *MASP1* (log2 FC = 0.944; adj *p* = 0.380*),* whose mRNA expression was found upregulated in a separate study conducted using frontal lobe post-mortem brains from MSA patients and controls [36].

### Collagen genes are upregulated in MSA

In the combined MSA group after *p*-value combination we detected 35 genes, most of them upregulated in patients. In both enrichment analyses we detected a key role of collagen genes: *COL4A1*, *COL4A2*, and *ITGA11;* all upregulated. *COL4A1* (collagen type IV alpha 1 chain) and *COL4A2* (collagen type IV alpha 1 chain) encode respectively for the alpha 1 and alpha 2 chains of type IV collagen which are important components of the basement membrane in all tissues, especially blood vessels. *ITGA11* (Integrin Subunit Alpha 11) is functionally related as it is a collagen receptor. Mutations in *COL4A1* and *COL4A2* have been associated with sporadic brain small vessel disease [56] and porencephaly [12]. Recently Paiva et al. [51] found *COL4A2* upregulated in both A30P aSyn mice and dopaminergic neurons expressing A30P aSyn, suggesting a key role of collagen-related genes in α-synuclein induced toxicity. In the same study, they demonstrated a regulation of *COL4A2* expression by miR-29a-3p, known to target *COL4A4* mRNA. In a separate study the loss of miR-29a was correlated with increased levels of BACE1 and amyloid-β in sporadic Alzheimer’s Disease [34]. Finally, lack of collagen VI has been related to neurodegeneration in mice models [13], and its presence has been related to a neuroprotective role against β-Amyloid toxicity [15].

Beside the collagen related pathway, the top genes detected in the differential expression analysis were: *ACTN1* (Actinin Alpha 1), *EMP1* (Epithelial Membrane Protein 1), and *NFIL3* (Nuclear Factor, Interleukin 3 Regulated). Expression changes of *ACTN1* were associated with AD in hippocampus [29], whereas *NFIL3* was associated with neuroprotection in models of Amyotrophic Lateral Sclerosis [70]. EMP1 protein was also found upregulated in 5xFAD AD model [25].

### MSA-C shows a common transcriptional background with Alzheimer’s disease

We detected a large functional network in MSA-C patients that included *APP* and other AD-related genes: *PSEN1*, *CLU*, *ROCK2, EFNA1* and *DYRK1*. The module (M1) including these genes was enriched for amyloid-β metabolism. A significant enrichment between MSA-C and AD DEGs was found in the temporal cortex and parahippocampal gyrus (AMP-AD data), but not in the other 5 regions analyzed. Additionally, after intersecting the list of genes between MSA and AD (TCX and PHG), we found a total of 243 dysregulated genes that overlapped in temporal cortex, 166 in parahippocampal gyrus, and 126 shared between both regions and MSA-C. Our results suggest that AD and MSA may share a common transcriptional background.

AD is a neurodegenerative disorder clinically defined by gradual cognitive impairment and alterations in executive function. The symptoms are correlated to the loss of synaptic connections and overall neuronal cell death [11, 24, 71]. The neuropathological hallmarks of AD are the accumulation of amyloid-β plaques (Aβ) and neurofibrillary tau tangles (NFTs) [33]. AD and MSA do not share a common brain pathology, however, it is not unusual to observe the co-occurrence of synuclein, amyloid, and/or tau pathology and in fact several studies have focused on the potential role of α-synuclein in the pathophysiology of AD [71] and it has also been reported that α-synuclein inclusions are found in more than 50% of autopsy-confirmed AD cases [4, 32, 42]. Data from human and mouse cell cultures suggest a role of α-synuclein in the GSK3β-mediated phosphorylation of tau. Additionally, in vivo models suggest Aβ could increase GSK3β activity inducing tau phosphorylation as well as α-synuclein production, leading to a cycle that could produce more amyloid-β and hyperphosphorylated tau in the process [71]. The presence of dysregulated AD-related genes in the MSA brains in our study might also suggest an involvement of Aβ and/or tau species in MSA. It is possible that soluble amyloid-β species may play a role in MSA and therefore might not be manifest as insoluble plaques at autopsy. This could be due to aging effects (due to the typically younger age of onset in MSA patients compared to AD) or the location within the brain that is examined (cerebellum may demonstrate higher resistance to plaque and/or tangle formation compared to medial temporal lobe regions that typically demonstrate high plaque and tangle burden in AD patients). Nonetheless, the finding of gene expression overlap with AD-related genes in the MSA cerebellum is of interest from a mechanistic and perhaps even therapeutic level.

### Study limitations

We note some limitations of our study. First, MSA is a rare disease and although our cohort is the largest that has been expression profiled to date it is still likely that we are underpowered to detect small effect sizes that could be functionally important. Secondly, we acknowledge that the findings would be improved by the inclusion of additional brain regions that may be altered by the disease. For example, it is not particularly surprising that we noted the most significant cerebellar transcriptional changes in MSA-C, a clinical subtype of MSA with predominating cerebellar symptoms. It would be interesting to compare the transcriptomic changes in the striatum, olivary nuclei, and pontine nuclei as well. Thirdly, we assessed C1 and C2 using different profiling approaches. This could be considered a positive aspect of our work as the identified transcriptional changes that cross-validate are likely not specific to a particular gene expression measurement approach and therefore may have higher reproducibility, however, this could also be considered a limitation as some true associations may be unreported due to their failure in one of the profiling chemistries and not due to the underlying biology. Additionally, layering additional genomic information – like DNA sequence information – would also enhance the study as it could facilitate more detailed analyses such as allele specific expression or epigenetic regulation of transcription. Finally, the samples from C1 were made up of clinical diagnoses only, not allowing us to perform comparisons between the neuropathological subtypes (OPCA and SND) as we were able to do for samples from C2.

## Conclusions

This is the largest study ever conducted on the MSA brain transcriptome. We utilized two different cohorts that were each assessed by different gene expression analysis chemistries that we propose increases the robustness of DEG and co-expression network detection.

The main findings of this study are the multiple evidence of oligodendrocyte gene downregulation associated with the loss of myelination. We detected the *QKI* gene as a master regulator of this associated gene network. Additionally, we showed an upregulation of neuronal-specific gene expression possibly as a consequence of the initial accumulation of monomeric α-synuclein in neurons, with *TIAM1* and *SYNGAP1* as top hubs in the two networks. An additional coexpression network highlighted the later stages of the neurodegenerative cascade with activation of microglia and astrocytes. Finally, our results suggest a common transcriptional background between MSA and AD, potentially through *APP*-mediated mechanisms.

## Supplementary information

**Additional file 1 Supplementary Appendix. Supplementary Methods. Supplementary Results. Figure S1.** PCA analysis conducted on cohort 1 after transformation of the raw counts using regularized log2 transformation. Samples were labeled by diagnostic status. No significant outliers were detected. The final sample size was: MSA = 19, HC = 19. **Figure S2.** PCA analysis conducted on cohort 2 after transformation of the raw counts using regularized log2 transformation. Samples were labeled by diagnostic status. One outlier was identified (A) and removed (B). The final sample size was: MSA = 47; HC = 47. **Figure S3.** PCA analysis conducted on oligodendrocytes LCM data from cohort 1 after transformation of the raw counts using regularized log2 transformation. Samples were labeled by diagnostic status. Three extreme outliers were identified (A) and removed (B). The final sample size was: MSA = 4; HC = 5. **Figure S4.** Number of differentially expressed genes (adj *p* < 0.05) detected in cohort 1 and cohort 2 for each MSA subtype (and in the analysis MSA-C + MSA-P, indicated as “MSA”, as well as in the *p*-value combination analysis (CB). The barplots are colored in red and blue for the number of upregulated and downregulated genes, respectively. We observed the larger number of DEGs in the MSA-C subtype in both cohort specific and combined analyses. **Figure S5**. The log2 FC between cohort 1 and 2 was compared using the *Spearman* correlation. In all the comparisons (MSA, MSA-P and MSA-C) we obtained a positive and statistically significant correlation (*ρ* > 0.204; *p* < 2.2E-16). In red genes with the same log2 Fold Change trend. **Figure S6.** Volcano plot reporting the differential analysis between SND (top) and OPCA (bottom) vs HC in C2. We detected a total of 7 and 58 genes, respectively. **Figure S7.** Log2 FC Correlation plots between MSA-P **vs HC** and SND **vs HC** (C2) (top), and between MSA-C **vs HC** and OPCA **vs HC** (bottom). We detected only one gene significant in both MSA-P/SND **vs HC** and 47 in both MSA-C/OPCA **vs HC** analyses (red dots). **Figure S8.** Results of the functional module discovery analysis using the 35 DEGs identified in MSA (MSA-P + MSA-C). The module M1 is enriched for cell-cell adhesion processes, and the module 2 is enriched for angiogenesis (*q* < 0.01). **Figure S9.** Protein-protein network generated using the prioritization method using as input all the DEGs detected in MSA-C after *p*-value combination. The analysis was conducted using *WEBGESTALT* [21]. The hub gene *APP* is the large circle. **In red and blue upregulated and downregulated genes in MSA-C, respectively. Figure S10.** Top 20 GO enrichment functional class detected using all the differentially expressed genes detected in MSA-C. We detected a total of 625 significant functional classes (adj *p* < 0.05). **Figure S11. Volcano plot reporting the differential analysis between OPCA vs SND in C2. We detected a total of 156 DEGs. Figure S12. Enrichment of AD genes in MSA-C dataset. Figure S13.** Results of the functional module discovery analysis using the 187 DEGs identified in LCM oligodendrocytes (MSA vs HC). The table showed below reports the GO top 3 significant enriched processes for each module. **Figure S14.** GO enrichment analysis results obtained using the LCM DEGs. We detected an enrichment for myelination processes, especially due to downregulated genes in MSA. **Figure S15.** Hypergeometric test results of the cell specific genes classification. The number at the top indicates total number of genes for each class. Dashed line indicates statistical significance (adj *p* < 0.05). We detected a significant enrichment for astrocyte (A) and oligodendrocyte (O) genes. **Figure S16.** Results of the gene classification by cell type using our deconvolution method, including DEGs with adj *p* < 0.10 (A), and DEGs with adj *p* < 0.025 (B). These results confirm what we obtained using a more restrictive cutoff (adj *p* < 0.05; Fig. 4). **Figure S17.** WGCNA analysis: Scale-free fit index (y-axis) as a function of the soft-thresholding power (x-axis). We selected “9” as soft threshold power for the WGCNA analysis. **Figure S18.** Dendrogram and heatmap showing the correlation between coexpression modules. **Figure S19.** Module membership (x-axis) vs Gene-trait significance (y-axis) for the yellow and green modules, significantly associated with MSA-C. **Figure S20.** Module membership (x-axis) vs Gene-trait significance (y-axis) for the blue and brown modules, significantly associated with MSA-C. **Figure S21.** Dot plot representing the top 15 GO process enriched in the four coexpression modules significantly different between MSA-C and HC. **Figure S22.** Cell specific enrichment among the WGCNA modules associated with MSA-C in cohort 1. We observed significant enrichment (labeled with a star) of: 1. astrocyte (adj *p* = 3.3E-19) and endothelial genes (adj *p* = 2.8E-04) in the yellow module; 2. neuronal genes in the brown module (adj *p* = 2.5E-60); 3. oligodendrocyte genes in the blue module (adj *p* = 7.7E-33). **Figure S23.** Plot representing the *Zsummary* statistics (x-axis) and the module size (y-axis). The *Zsummary* statistics indicates the preservation of a module in a test dataset. In this case, we are investigating whether the modules detected in C1 are preserved in C2. Focusing on the modules associated with the diagnostic status, the *Zsummary* statistics indicates a strong preservation for blue and brown module and a moderate preservation for the green module. No evidence of preservation was found for the yellow module. The gold module represent a random set of genes across the dataset. **Figure S24. Comparison between differentially expressed genes from the QKI-KO study from Darbelli et al. (2017) and genes detected in the QKI module, dowregulated in MSA-C, enriched for both oligodendrocyte genes and myelination processes.**

**Additional file 2 Table S1**. Differentially expressed genes detected in the cohort 1 and cothort 2 in MSA. **Table S2**. Differentially expressed genes detected in the cohort 1 and cothort 2 in MSA-P. **Table S3**. Differentially expressed genes detected in the MSA-C in cohort 1 and 2 ranked by *p*-value. **Table S4**: Complete significant Results of the *p*-value combination for MSA (MSA-P + MSA-C). **Table S5**: Complete significant results of the *p*-value combination for MSA-P. **Table S6**: Complete significant results after *p*-value combination of the differential expression results from the two MSA-C cohorts. **Table S7.** Differentially expressed genes in SND (A) and OPCA (B) MSA patients from cohort 2. Genes significant also stratifying by the corresponding clinical subtype are reported in grey (MSA-P/SND or MSA-C/OPCA). **Table S8**. Complete results of the functional network enrichment analysis for the DEGs detected in MSA-C. **Table S9.** Differentially expressed genes detected in the comparison MSA-C vs MSA-P in cohort 1. No differentially expressed genes were detected in cohort 2. **Table S10**: Top 10 results after *p*-value combination of the differential expression results (MSA-C vs MSA-P) from the two MSA-C cohorts. No genes showed significance after multiple test correction. **Table S11.** Differentially expressed genes from the comparison OPCA vs SND patients from cohort 2. **Table S12**. Results of the GO analysis from the 156 DEGs obtained in the comparison OPCA vs SND. **Table S13**. Enrichment of AD genes (AMP-AD datasets) among MSA-C Differentially expressed genes. (A) Enrichment analysis run using different DEGs cutoff to select AD genes. (B) Enrichment analysis run using non-DEGs (adj *p* ≥ 0.950). **Table S14**. Overlap between MSA-C DEGs, AD temporal cortex and AD parahippocampal gyrus (data from AMP-AD). Genes are ranked by adj *p* in MSA-C. **Table S15.** Differentially expressed genes detected in oligodendrocytes (adj *p* < 0.05). **Table S16**. Results of the Cell Specific GO analysis. **Table S17**. Details of the genes included in the color-coded modules significantly associated with MSA-C. The genes are ranked for Module Membership *p*-value. Hub genes for each module are indicated in bold. **Table S18**. Complete results of the enrichment analysis conducted on the 4 co-expression modules associated with MSA-C. The GO categories are ranked by *p*-value. **Table S19**: Comparison between the differentially expressed genes obtained in the study from Darbelli et al. (2017) involving mice with excised QKI-exon 2 (QKI deficient) versus QKI-proficient.
